# Disturbances in Electrodermal Activity Recordings Due to Different Noises in the Environment

**DOI:** 10.3390/s24165434

**Published:** 2024-08-22

**Authors:** Dindar S. Bari, Haval Y. Y. Aldosky, Christian Tronstad, Ørjan G. Martinsen

**Affiliations:** 1 Scientific Research Center, University of Zakho, Zakho 42002, Iraq; dindar.bari@uoz.edu.krd; 2 Department of Physics, College of Science, University of Zakho, Zakho 42002, Iraq; 3 Department of Physics, College of Science, University of Duhok, Duhok 99454, Iraq; 4 Department of Clinical and Biomedical Engineering, Oslo University Hospital, 0424 Oslo, Norway; 5 Department of Physics, University of Oslo, 0371 Oslo, Norway

**Keywords:** electrodermal activity, electrodermal responses, noise, skin, sweat, wearable

## Abstract

Electrodermal activity (EDA) is a widely used psychophysiological measurement in laboratory-based studies. In recent times, these measurements have seen a transfer from the laboratory to wearable devices due to the simplicity of EDA measurement as well as modern electronics. However, proper conditions for EDA measurement are recommended once wearable devices are used, and the ambient conditions may influence such measurements. It is not completely known how different types of ambient noise impact EDA measurement and how this translates to wearable EDA measurement. Therefore, this study explored the effects of various noise disturbances on the generation of EDA responses using a system for the simultaneous recording of all measures of EDA, i.e., skin conductance responses (SCRs), skin susceptance responses (SSRs), and skin potential responses (SPRs), at the same skin site. The SCRs, SSRs, and SPRs due to five types of noise stimuli at different sound pressure levels (70, 75, 80, 85, and 90 dB) were measured from 40 participants. The obtained results showed that EDA responses were generated at all levels and that the EDA response magnitudes were significantly (*p* < 0.001) influenced by the increasing noise levels. Different types of environmental noise may elicit EDA responses and influence wearable recordings outside the laboratory, where such noises are more likely than in standardized laboratory tests. Depending on the application, it is recommended to prevent these types of unwanted variation, presenting a challenge for the quality of wearable EDA measurement in real-world conditions. Future developments to shorten the quality gap between standardized laboratory-based and wearable EDA measurements may include adding microphone sensors and algorithms to detect, classify, and process the noise-related EDA.

## 1. Introduction

Electrodermal activity (EDA) refers to the variations in the electrical properties of the skin that arise from the sweat glands’ activity. The activation of the sweat glands leads to the filling of the sweat ducts, which consequently causes a swift increase in the skin conductance via the generation of paths of ionic current through the dry and highly resistive layer of the skin called the stratum corneum. EDA is widely employed in psychophysiological measurements due to its strong association with the activity of the sympathetic nervous system. In addition, EDA is frequently used in studies related to stress and emotion since the sympathetic nervous system is affected by the hypothalamus and limbic system, which are parts of the brain that deal with emotional aspects [1].

EDA as a biomarker is widely used in psychophysiological research such as emotion and cognition studies. However, EDA signals can be influenced by several factors, which should be avoided during recording unless they are intended to be included in the study. For example, EDA responses can be affected by some environmental conditions, such as the environmental temperature and humidity [1]. In addition to the environmental conditions, EDA can also be affected by other external factors, such as noise (high sound levels) [2]. Therefore, EDA is a very sensitive measurement for many sources of stimuli, but this implies that the measurement has low specificity for inference in targeted factors unless other influencing factors are controlled, which is increasingly difficult outside of the laboratory.

In the majority of previously conducted research, EDA signals have been recorded in laboratory environments, where participants are usually seated in a controlled condition. However, in recent applications of EDA, signals are more often recorded in uncontrolled environments (i.e., outside laboratories), which may influence the recordings. Notably, a variety of wearable instruments have been developed to enable the possibility of recording EDA signals in real-world situations. Malathi et al. [3], for example, proposed a wearable EDA device for the detection of drowsiness in drivers. Leite et al. [4] used a wearable EDA wireless sensor to investigate the affective states of children when interacting with robots in daily situations. Kim and Fesenmaier [5] utilized a wearable EDA tool to assess travelers’ emotions in real time (natural settings) throughout a four-day visit. Thammasan et al. [6] employed a wrist-worn EDA sensor for the purpose of monitoring physiological signals in a school environment. Nasseri et al. [7] used some commercially available wearable sensors to record various physiological signals, including EDA, from patients with epilepsy in the hospital or at home. Vieluf et al. [8] operated a wearable EDA device to measure EDA waveforms in patients with and without seizures over 24 h. Zhu et al. [9] used a wrist-based EDA device to predict people’s stress status.

Among the most significant issues that may influence EDA signals when employing EDA devices in daily life or clinical applications could be noise pollution (high sound pressure). However, the degree of noise influence in different EDA recording cases is largely unknown, and controlling noise exposure is often impractical—for instance, in the long-term recording of EDA during daily life. Noise can cause interference during data collection, which may affect individuals’ physiological responses and therefore produce artifacts. Therefore, ensuring the quality of EDA recordings in uncontrolled environmental conditions represents a challenge. 

The influences of environmental noise on SC and SP are discussed in very few studies. Masullo et al. [2], for instance, recorded SC following three different levels of noise (60 s long), which were low at 70 dB, medium at 78 dB, and a high level at 87 dB. The authors found that the SC increased as the noise level increased. Park et al. [10] investigated changes in SC due to floor impact noise and road traffic noise among two subject groups with low and high noise sensitivity. The researchers reported that the SC increased as a result of noise exposure with a duration of 5 min. Uno and Grinkgs [11], with stimulus durations of 2 s, and Raskin et al. [12] also studied the effects of the sound stimulus intensity and repetition on both the SC and SP and observed that the SC and SP significantly increased as the stimulus intensity increased.

However, these studies were concerned with only one or two EDA parameters (SC and/or SP) and a few types and levels of noise. Therefore, it is unknown to what extent the different sound noises in the environment affect all EDA measurements, particularly when more than one EDA parameter is simultaneously measured following various types and levels of noise. In addition, we aimed to investigate (through laboratory experiments) whether and how sounds from various sources of the surrounding environment may affect each EDA parameter. This is generally relevant when wearable EDA devices are employed and when the experimental conditions are considered for the planning of studies utilizing EDA measurements, and also in comparison among various studies. The aim of this study was to recognize the influence of various noise disturbances on three (SC, SS, and SP) EDA parameters simultaneously at the same skin site, by utilizing a novel EDA recording device.

The rest of the paper is arranged as follows. In Section 2, the materials and equipment used, the experimental protocol, the data, and the statistical analysis are introduced. In Section 3, the results are provided. In Section 4, a discussion of the results is provided. Finally, in Section 5, the conclusions are summarized.

## 2. Materials and Methods

In this study, a new EDA recording system was used, which was capable of recording three EDA (SC, SS, and SP) parameters simultaneously at the same skin site. The system consisted of a front-end electronic box designed for connection to a National Instruments data acquisition (DAQ) card that was interfaced with a laptop running software written in LabVIEW, v. 14®.

The employed technique was a three-electrode system consisting of one measuring electrode, one reference electrode, and a current sink electrode. The SP was measured by recording the DC voltage between the measuring electrode and the reference electrode. Simultaneously, both the reference electrode and the current sink electrode were used to provide SC and SS measurements beneath the measuring electrode, as described in the literature.

In order to provide the AC current, a Howland current pump was used. A 200 mV voltage was generated by the PC and fed to the Howland circuit via the DAQ system. The Howland circuit (front-end electronic box) in turn converted the input voltage (200 mV) to about 20 μA AC (20 Hz) and delivered it to the skin via the measuring electrode.

**Figure A1:**
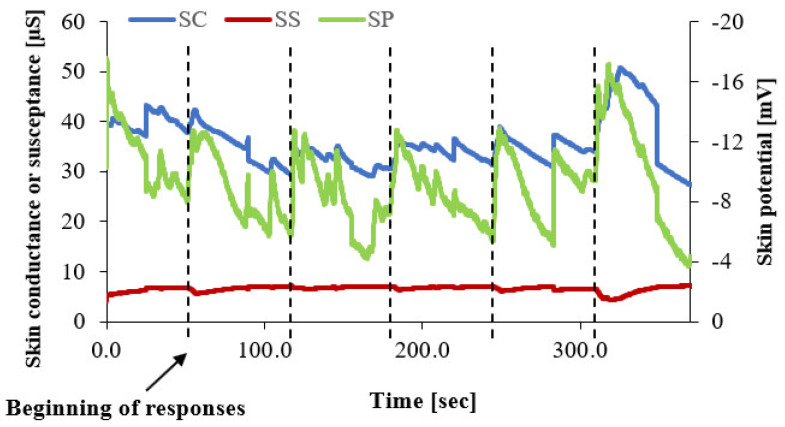
A typical EDA waveform recorded from a participant shows variations in EDA responses following noise stimuli. SC = skin conductance, SS = skin susceptance, and SP = skin potential.

Analog signals were received back from the skin via the electronic front-end electronic box and converted to digital form by a DAQ card. The digitized signals were then processed by differentiation in LabVIEW and separated into a DC component for SP measurement and an AC component for SC and SS measurement by utilizing a conventional lock-in technique. Typical EDA waveforms recorded from a participant are shown in Figure A1.

### 2.1. Participants and Experimental Protocol

A group of forty healthy adults (20 male and 20 female, mean age: 23.6 years) were recruited primarily from the student population at the University of Zakho and gave written informed consent prior to participation and after fully understanding the protocol of the study. The participants were asked whether they had any hearing problems or had any problems with the high sound levels for their safety, as well as the quality of the recorded EDA data (i.e., normal hearing was required for all participants). During the measurements, all participants were seated comfortably in a chair and they were not allowed to speak with the operators unless absolutely necessary. All EDA measurements were conducted at the University of Zakho in a confined laboratory setting at a specified temperature range (22–23 °C). The study was approved by the Research Ethics Committee at the University of Zakho.

**Figure 1:**
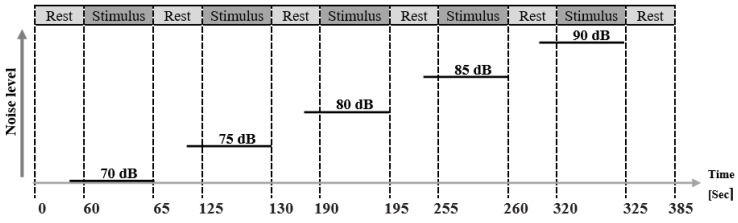
Typical timeline for the EDA recordings.

Once the participants had finished reading the information sheet regarding the experiment and signed the informed consent form, three solid-gel ECG electrodes (Kendall Kittycat 1050NPSM Neonatal Electrodes, Medtronic, Minneapolis, MN, USA) were placed on one arm of each participant. The first electrode was positioned on the hypothenar site of the palm, the second on the apex of the elbow, and the third one was placed on the underarm between the first and second electrodes, as illustrated in Bari et al. [13]. Before the EDA measurements started, at least 5 min were allowed for electrode stabilization. In order to investigate the effects of various noises on EDA, each participant was exposed to five different noise types and noise levels, as indicated in Figure 1. The five selected noise (low-frequency sound) stimuli were (1) house electric fan noise (70 dB); (2) industrial noise (75 dB); (3) an adult’s walking noise (80 dB); (4) intensive care room unit noise (85 dB); and (5) ambulance siren noise (90 dB).

All noise stimuli were edited to have a duration of 5 s. After each audio stimulus and before playing the next audio stimulus, there was a relaxation period of 60 s, waiting for the EDA waveform to become stable again at the new baseline. The recording took 385 s in total. Noise stimuli were played over a loudspeaker situated approximately 1 m in front of the participants. The loudspeaker was connected to a PC, which controlled the audio playback system and sequentially played the noise stimuli via PowerPoint slides. Each stimulus was presented once for each of the 5 stimuli.

### 2.2. Data and Statistical Analysis

In order to extract all EDA response features, the onsets and peaks of the specific EDA responses were identified. The features were the time from the onset of SCRs to peak SCRs (SCRs_Tris), the skin potential relative early turn (SPRET), the amplitude of the skin conductance response (SCRs_Amp), the amplitude of the skin potential response (SPRs_Amp), and the amplitude of the skin susceptance response (SSRs_Amp).

**Figure 2:**
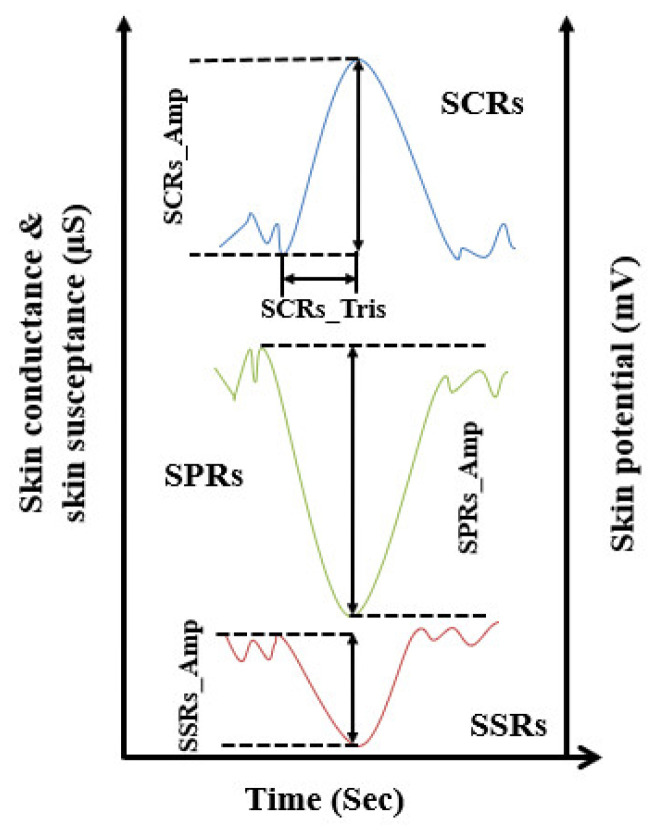
Examples to illustrate how the onsets, peaks, and amplitudes of the SCRs, SSRs, and SPRs are specified.

SCRs_Amp, SPRs_Amp, and SSRs_Amp were calculated from the differences between the onsets and peaks of SCRs, SPRs, and SSRs as shown in Figure 2. In addition, for monophasic SPRs, SPRs_Amp was calculated from the onsets to the (positive/negative) peaks of the SPR waveforms, whereas, for biphasic SPRs, SPRs_Amp was computed from the first (negative/positive) peak to the second (positive/negative) peak [1]. SCRs_Tris was computed from the time interval between the onset and the peaks of the SCRs. Finally, the SPRET was obtained by subtracting the SCR peak time from the SPR peak time, dividing the result by the SCR onset to peak time and multiplying the result by 100%.

Statistical analyses were carried out using SPSS Statistics (version 22). The main effects of the different noises on the EDA responses were assessed using a repeated-measures analysis of variance (ANOVA). In addition, a post hoc multiple pairwise comparisons test using the Sidak correction was utilized in order to compare groups (different levels of noise) against each other. *p* values of less than 5% were considered as statistically significant.

The effects of the noise level on the EDA waveforms were also statistically evaluated by a linear mixed effects model, using either SCR_Amp, SPR_Amp, SSR_Amp, SCRs_Tris, or SPRET as a dependent variable with respect to the noise level as a fixed effect, with the subject as a random effect, including the random slope and intercept in the model. The analysis was performed with the fitlme() function in MATLAB, version 2015a (in the statistics and machine learning toolbox).

## 3. Results

**Figure 3:**
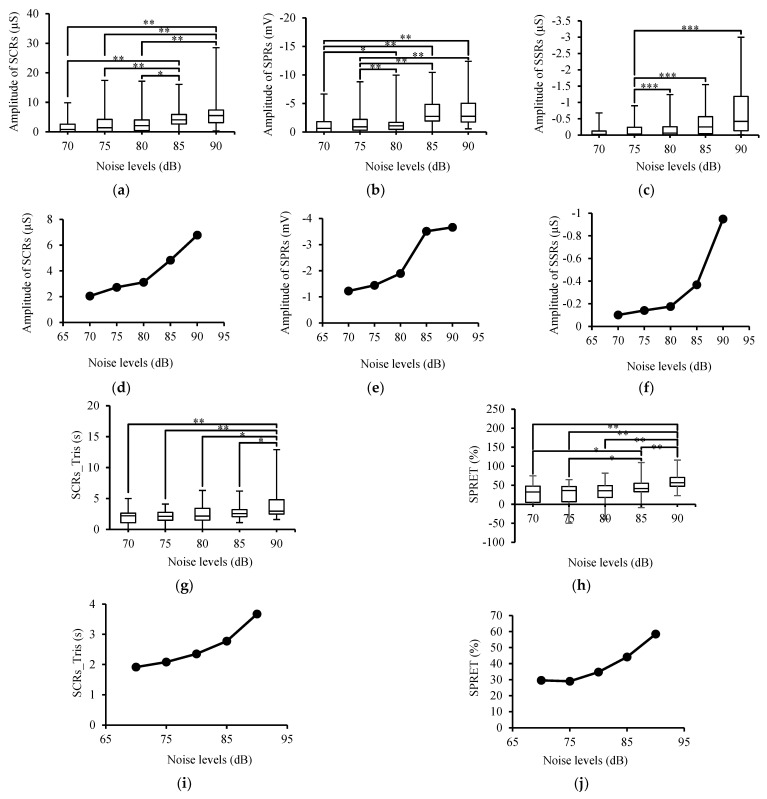
(**a**) SCRs_Amp, * *p* < 0.05 and ** *p* < 0.005; (**b**) SPRs_Amp, * *p* < 0.05 and ** *p* < 0.005; and (**c**) SSRs_Amp, *** *p* < 0.001 as a function of noise level; relation between noise levels and (**d**) mean values of SCRs_Amp, 95% CI from 0.8 to 1.5; (**e**) mean values of SPRs_Amp, 95% CI from −0.891 to −0.498; (**f**) SSRs_Amp, 95% CI from −0.320 to −0.063; (**g**) SCRs_Tris as a function of noise level, * *p* < 0.01 and ** *p* < 0.001; (**h**) SPRET as a function of noise level, * *p* < 0.01 and ** *p* < 0.001; relation between noise levels and (**i**) mean values of SCRs_Tris and noise level, 95% CI from 0.214 to 0.628; (**j**) mean values of SPRET and noise level, 95% CI from 4.914 to 9.677. In all boxplots, whiskers represent the maximum and minimum of the data.

Figure 3a shows SCRs_Amp with respect to the different noise stimuli levels. It was found that SCRs_Amp increased with the increasing noise levels (from the different types of noise). The results of the repeated-measures ANOVA confirm that the effect of the noise level on SCRs_Amp was statistically significant (*p* < 0.001). Moreover, the post hoc pairwise multiple comparison tests also indicated significant differences among the groups (different noises), in which the noise at 85 and 90 dB was significantly (*p* < 0.005) different from the other levels, as indicated in Figure 3a.

Figure 3d shows that SCRs_Amp increased with the different noise levels, reaching a maximum value at 90 dB. In addition, the statistical analysis with the linear mixed effects model revealed that the noise level had a significant effect (*p* < 0.001) on SCRs_Amp, increasing by roughly 1.2 μS per 5dB as estimated in the linear model (95% CI from 0.8 to 1.5).

As shown in Figure 3b, the median values of SPRs_Amp increased in a negative trend following the higher noise level. In addition, the results of the repeated-measures ANOVA revealed that noise had a significant (*p* < 0.001) effect on SPRs_Amp. Moreover, the pairwise post hoc testing identified significant differences among the noise levels, and the noise levels of 85 and 90 dB were highly different (Figure 3b) from the rest of the groups.

The average (over 40 participants) of SPR_Amp consistently showed a stepwise increase with the increasing noise level, as seen in Figure 3e. On average, the experienced noise-induced SPR_Amp increased with the increasing noise level, reaching a maximum value at 90 dB. Furthermore, the results of the linear mixed effects model showed that SPRs_Amp significantly (*p* < 0.001) increased in the negative direction with approximately −0.7 mV per 5dB, as estimated in the linear model (95% CI from −0.891 to −0.498).

The results of SSRs_Amp are plotted in Figure 3c. It can be clearly seen that SSRs_Amp changed as a function of the noise level. The differences among the noise level data were statistically significant (*p* < 0.001), as indicated by the ANOVA analysis. The pairwise comparison tests showed that the differences among the noise groups were statistically significant, and SSRs_Amp at a noise level of 75 dB was significantly (*p* < 0.001) different from that in the other groups, except for the data at a noise level of 70 dB.

It is of note (Figure 3f) that SSRs_Amp increased significantly (*p* > 0.005) in the negative direction by about −0.2 μS per 5dB, as estimated in the linear model (95% CI from −0.320 to −0.063).

Based on the findings presented in Figure 3g,i, there was an association between SCRs_Tris and the noise level. The ANOVA analysis of SCRs_Tris demonstrated significant (*p* < 0.001) differences among the groups (noise levels), and pairwise comparisons among the noise levels were performed with Sidak post hoc pairwise comparison tests, indicating significant differences between noise at the level of 90 dB and noise at the rest of the levels, as shown in Figure 3g.

It is clear from Figure 3i and the results of the linear mixed effects model that the noise level had a significant (*p* < 0.001) linear effect on SCRs_Tris, increasing by 0.4 s per 5dB, as estimated in the linear model (95% CI from 0.214 to 0.628).

Figure 3h shows the SPRET for all participants and indicates that an increase in the SPRET coincides with increases in the noise level. The ANOVA test confirmed that the SPRET was significantly (*p* < 0.01) influenced by the increase in the noise level. The pairwise comparison tests showed significant differences among the groups, particularly between the groups of 85 and 95 dB and the other groups, as noted in the figure.

Interestingly, in Figure 3j, it can be seen how the SPRET increased with the noise level. Furthermore, the analysis of the linear mixed effects model confirmed that the SPRET was significantly (*p* < 0.001) affected by the increase in the noise level, increasing by 7.3% per 5dB, as estimated in the linear model (95% CI from 4.914 to 9.677).

## 4. Discussion

The purpose of the present laboratory-based study was to highlight the potential effects of different noises (noise types and levels) in the environment on EDA measurements. Generally, the findings of this study emphasize that the different brief noises at various levels led to significant changes in the EDA recordings. The three EDA phasic parameters (SCRs, SPRs, and SSRs) were significantly increased with increasing noise levels, which was due to higher defense/startle responses (reactions) to more intense noises [14].

The results from this laboratory experiment reveal that the intensity of different ambient noises has a significant effect on SCRs_Amp. The SCRs_Amp values increased as a result of the different noises. The increases in SCRs_Amp were due to the fact that stronger noises lead to stronger sympathetic nervous system responses, which then cause sweat gland activation. As a result, higher SCRs_Amp values occur due to sweat duct filling and secretion [15]. This implies that participants experience stronger arousal from stronger sound levels based on the model of the relationship between the arousal intensity and physiological responses [16]. These findings are consistent with previous studies [2,10,11], reporting that noise significantly affects SCRs. Indeed, as the noise levels increase, the SCRs increase; hence, any SCRs recorded in a noisy environment may produce some additional SCRs that are not associated with the specific or unspecific SCRs of interest.

The effects of high ambient noise levels on SPRs_Amp were clearly seen in the present laboratory-based study. For all participants, SPRs_Amp was increased and reached a plateau (Figure 3e) due to sweating and skin hydration, interpreted as follows. Based on the voltage divider model [17], prior to noise stimuli, the SP is assumed to be low due to the high resistance through the sweat ducts, which gives access to the more negative potential at deeper layers. Following noise stimuli and sweat duct filling, the SP at the skin surface becomes more negative because the electrical resistance through the duct declines (i.e., the voltage drops as the ductal resistor decreases) with the filling of sweat [17]. These results are in agreement with Uno and Grinkgs [11] and Raskin et al. [12], who also found that SPRs_Amp was linearly related to the stimulus intensity, without mentioning any specific reason.

The impact of ambient noise on the third EDA response parameter (SSRs_Amp) has not previously been tested. We did not find a single study in the literature that investigated the effects of high sound stimuli or noise on SSRs in order to compare with our findings. The increases in SSRs_Amp as a consequence of noise stimuli are due to the hydration of the stratum corneum and an increase in the electrical capacitance of the skin. In addition, when the sudomotor nerves are activated, the eccrine sweat glands start sweating, and, as a result, the corneum is moisturized, mainly by the diffusion of sweat from the ducts into this layer. Moreover, in a previous study, it was proposed that the sweat ducts are not capacitive and so the SS is not related to the filling of the sweat ducts, like the SC and SP [18]. However, in several studies [15,19,20], the results of SSRs are reported, which could be due to variations in skin moisturization following sudomotor responses.

Significant impacts of noise on SCRs_Tris (time component of SCRs) were also noticed in this study. Based on the results of the linear mixed effects model, noise had a significant positive effect on SCRs_Tris, equivalent to 0.4 s per dB in a linear estimation. These findings are associated with the results of SCRs_Amp, as a stronger SCRs_Tris was related to a stronger SCRs_Amp because stronger SCRs usually take a longer time to reach their maximum.

The final EDA score analyzed in this study was the SPRET, which is related to the relative time difference between the peaks of the SPR and SCR waveforms. We wanted to test whether high noise levels could cause variations in this temporal distance between the SPR and SCR peaks (i.e., SCRs_Amp and SPRs_Amp). Figure 3h,j show that the SPRET had a monotonic relationship (increasing by a magnitude of 7.3% per noise level) with the noise intensity and the highest SPRET value was associated the highest noise level (90 dB).

In summary, this laboratory-based study shows the significant impacts of noise exposure on the generation of EDA responses dependent on the noise (type and level of noise). This clearly indicates that the participants experienced auditory arousal due to the sound stimuli. Although this study focused on short noise effects (5 s duration) and we did not consider the effects of longer stimuli on EDA responses, these unwanted noise effects could produce artifacts in EDA recordings, either within hospitals or in wearable situations. Therefore, EDA signals may benefit from filtering such noise artifacts once these responses are recorded in the field (i.e., outside of the laboratory), depending on the focus or application of the recording. 

Moreover, considering the finding of this study of the significant impact of typical environmental noise on EDA, this may be a confounding factor in some wearable applications of EDA, where it can be difficult to distinguish between, for instance, elevated stress levels and specific responses to noise without any contextual information. We, therefore, speculate as to whether some wearable applications of EDA can benefit from sensors for noise detection, allowing the identification of electrodermal responses (EDRs) caused by noise to be excluded to improve the accuracy of the calculated metrics. Smartwatches have already been found to provide reliable continuous noise monitoring in recreational and occupational environments [21], supporting the feasibility of this idea. In addition to simply detecting the noise level, signal processing and machine learning can provide the opportunity to classify different types of environmental sound using wearable devices, as shown by Jain et al. [22]. Adding such classification may further improve the automated filtering of wearable EDA events and, given sufficient data, may also provide an opportunity to learn which types of environmental noise have the most influence or are most impactful at the individual level. We, therefore, suggest future work to evaluate the potential benefits of adding sensor-based environmental noise-based EDA filtering to wearable devices and to estimate the performance improvement for either EDA-based event detection applications (such as detecting pain in non-verbal persons) or long-term state estimation applications such as stress monitoring. Initially, a laboratory study that combines both factors of interest (such as stress or pain) and disturbances (environmental noise) in the same experiment may provide data to calculate the EDA metric performance with vs. without event filtering. 

As an alternative approach beyond labeling discrete responses, we envision the possible implementation of noise-elicited EDA near-real-time correction for wearable devices as follows. Assuming a simple model of the measured EDA as the sum of intrinsic EDA and noise-elicited EDA, an estimation of the magnitude of the noise-elicited component can be subtracted from the intrinsic EDA score within practical time bins (for example, the sum of the skin conductance response amplitudes minus the sum of the estimated noise-elicited amplitudes within a 5 min window). The estimation of the noise-elicited amplitude can be based on the fit between the EDR amplitude and noise level (in dB), as in Figure 3d–f, indicating a possible exponential relationship to be used as a model function based on the shapes of the plots.

To perform benchmark comparisons within a larger sample size, future work might focus on investigating parameter optimization approaches for noise sources and conducting a comprehensive comparison between them. Subsequent research should examine the efficacy of this method in analyzing data derived from wearable sensors, where noise reduction is an essential first step. 

### Limit of the Study

In this study, only the effects of short noise stimuli (5 s) on EDA are investigated, and a further study using longer exposures (long-term static background noise) would be helpful in understanding long-term variations in EDA levels or responses due to different environmental static noise. Another limitation may be that the order of the noise stimuli was the same (i.e., not in a random fashion) for all subjects, which may have caused habituation with a stepwise increase in the noise levels. However, the positive correlations between the noise levels and EDA responses indicate that the effect of the noise level is significantly stronger than the habituation effect. Finally, another point to be considered as a limitation is that there was only one noise level (in dB) per type of noise, implying that the impact of the noise level could not be analyzed separately from the type of noise. However, the main reason that we chose different sounds was to avoid habituation, where the repetition of the same sound (five times) would likely lead to habituation.

## 5. Conclusions

The present study investigated the impacts of various ambient noises on EDA (psychophysiological) responses. As the results showed, changes in the three EDA parameters were found to depend on the type and/or level of noise, and, as the noise levels increased, the SCRs, SSRs, and SPRs increased. This means that short or sudden typical environmental noises can cause sudomotor responses, producing variations in EDA responses that may be regarded as artifacts. Therefore, it may be desirable to prevent these types of unwanted variation in some EDA applications, although this is rarely possible in wearable measurements in daily life. For this reason, wearable EDA devices may benefit from adding sensors to provide contextual information on these sources of EDA variation—for example, with noise sensors (microphones) or a smartwatch-based assistive device [21,23]. Moreover, when EDA signals are also impacted by the type of noise, the device may need to classify the type of noise, in addition to the intensity, to provide relevant contextual information. It is feasible to greatly improve the accuracy and reliability of wearable EDA measurements in real-world settings through the use of multi-sensor technology, signal processing, machine learning, and user-centric approaches.

## Data Availability

The original contributions presented in the study are included in the article; further inquiries can be directed to the corresponding author.

## Appendix A

Figure A1 shows an example of the EDA waveforms recorded from a participant. It is clear from the figure that different sound stimuli cause different EDA responses (the beginning of the responses due to each sound stimulus are indicated by vertical lines). In addition, the highest EDA responses are associated with the strongest sound level.
